# Effective management of pulmonary embolism after initial diagnosis of acute coronary syndrome: a case report highlighting differential diagnosis challenges

**DOI:** 10.3389/fmed.2025.1518628

**Published:** 2025-04-02

**Authors:** Jiahuan Rao, Zhiyan Wang, Haibo Chen

**Affiliations:** ^1^Department of Cardiology, Shenzhen Second People’s Hospital, the First Affiliated Hospital of Shenzhen University, Shenzhen, China; ^2^Shenzhen Finance Institute, The Chinese University of Hong Kong, Shenzhen, China

**Keywords:** diagnosis, acute coronary syndrome, pulmonary embolism, chest pain, management

## Abstract

Pulmonary embolism (PE) often presents with symptoms similar to acute coronary syndrome (ACS), making diagnosis challenging. We report a case of a 55-year-old male with hypertension, chronic kidney disease, and hyperuricemia who developed chest pain and shortness of breath. Initial evaluation suggested ACS due to electrocardiogram changes and elevated cardiac biomarkers. However, coronary angiography (CAG) showed no significant stenosis, prompting further diagnostic workup. Computed tomography pulmonary angiography (CTPA) confirmed PE, likely secondary to deep vein thrombosis (DVT) in the right lower extremity. The patient was treated with an inferior vena cava (IVC) filter and thrombus aspiration, followed by anticoagulation therapy. This case highlights the critical need to differentiate PE from ACS and emphasizes the importance of a multidisciplinary approach in managing thromboembolic events to ensure optimal patient outcomes.

## Introduction

Chest pain is a common symptom in both emergency and outpatient settings, with a broad differential diagnosis that includes acute coronary syndrome (ACS) and pulmonary embolism (PE) (1). Correctly distinguishing between these conditions is critical, as misdiagnosis can lead to inappropriate management and poor patient outcomes (2). ACS and PE often present with overlapping symptoms such as chest pain and dyspnea, complicating the diagnostic process (3).

Acute coronary syndrome refers to a spectrum of conditions like unstable angina and myocardial infarction caused by acute myocardial ischemia, diagnosed through clinical assessment, electrocardiogram (ECG), and cardiac biomarkers (4). In contrast, PE results from obstruction of the pulmonary arteries, typically due to thrombi from deep vein thrombosis, and is diagnosed using imaging such as computed tomography pulmonary angiography (CTPA) (5, 6). This case report underscores the diagnostic challenges between ACS and PE, emphasizing the need for a thorough approach to ensure accurate and timely treatment.

## Case presentation

### Patient demographics and clinical characteristics

The patient is a 55-year-old male with a 10-year history of hypertension, chronic kidney disease, hyperuricemia, and gout. He has smoked and consumed alcohol for 30 years but has no family history of cardiovascular or genetic diseases.

Four days before his medical visit (July 10, 2024), during a football game, he experienced precordial chest pain, palpitations, and shortness of breath, relieved by rest. Minimal exertion, such as climbing stairs, consistently triggered these symptoms, leading him to seek care. Initial vitals: temperature 36.5°C, heart rate 80 bpm, respiratory rate 20 breaths/min, blood pressure 145/102 mmHg, oxygen saturation 98%, and blood glucose 5.8 mmol/L. His extremities were warm, without edema.

### Initial evaluation

**FIGURE 1:**
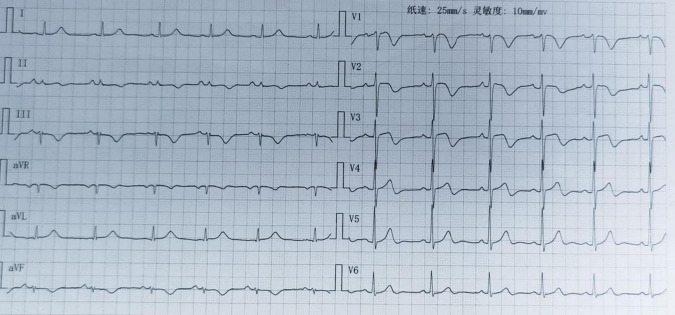
Electrocardiogram (ECG) of the patient upon admission. At the first medical contact, the electrocardiogram demonstrated ST-segment elevation in leads V1-V3 and T-wave inversions in leads II, III, and aVF.

**FIGURE 2:**
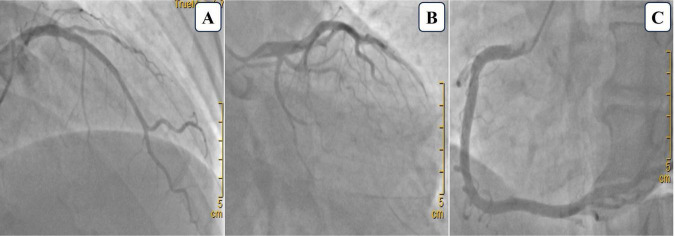
Coronary angiography images. **(A)** Cranial view showing 20% stenosis in the proximal to mid-segment of the left anterior descending artery. **(B)** Caudal view showing no significant stenosis in the left main artery and a small, non-stenotic left circumflex artery. **(C)** Left anterior oblique view of the right coronary artery demonstrating no stenosis.

Upon initial evaluation (July 14, 2024), the patient’s ECG showed ST-segment elevation in leads V1-V3 and T-wave inversions in leads II, III, and aVF (Figure 1). Echocardiogram revealed left ventricular segmental wall motion abnormalities, moderate tricuspid regurgitation, and estimated pulmonary artery systolic pressure of 56 mmHg. Lab tests showed troponin T (TNT) 0.022 μg/L, high-sensitivity troponin I (hs-TNI) 0.079 ng/ml, N-terminal pro-B-type natriuretic peptide (NT-proBNP) 3404 pg/ml, and D-dimer 4.13 mg/L, raising suspicion for ACS. Emergency coronary angiography (CAG) was promptly performed but revealed no significant coronary artery stenosis (Figure 2). He was admitted to the cardiac care unit (CCU) for further evaluation.

### Modified diagnosis

**FIGURE 3:**
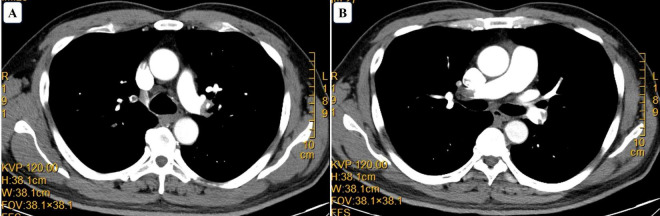
Computed tomography angiography. Computed tomography indicating pulmonary embolism. **(A)** Filling defect in the left main pulmonary artery. **(B)** Filling defect in the right main pulmonary artery.

On July 15, hs-TNI decreased to 0.052 ng/ml, NT-proBNP decreased to 1505 pg/ml, and D-dimer dropped to 2.57 mg/L. Repeat echocardiogram showed ventricular septal thickening and wall motion impairment. Chest CT revealed mild inflammatory changes in both lungs. Until now, the patient had a Wells score of 0 and a Geneva score of 3, both indicating a low pretest probability of PE. However, despite initial treatment for ACS, the patient continued to experience recurrent shortness of breath. Moreover, the elevated D-dimer levels remained unexplained, raising suspicion for an alternative diagnosis. Given the absence of significant coronary artery stenosis on CAG and the patient’s persistent symptoms, the clinical team decided to further investigate the possibility of PE. On July 16, venous ultrasound detected DVT in the right superficial femoral and popliteal veins. By July 17, CTPA confirmed extensive pulmonary emboli (Figure 3), leading to a definitive diagnosis of PE.

### Management and treatment

After the diagnosis of PE in the cardiology department, the patient immediately received multidisciplinary consultations. Based on the Pulmonary Embolism Severity Index (PESI) and simplified PESI (sPESI) (7, 8), the patient was classified as low risk for 30-day mortality (PESI class I and sPESI score of 0). Therefore, oral anticoagulation was recommended.

Due to the presence of DVT and to prevent further embolic events, an emergency inferior vena cava (IVC) filter was placed. Venography confirmed multiple filling defects in the right distal femoral and popliteal veins, leading to the successful insertion of the IVC filter just below the renal veins. Thrombus aspiration was performed via catheter-based thrombolysis targeting the right femoral and popliteal veins, yielding a large volume of dark red thrombus. A thrombolysis catheter was left in place for continued therapy.

Postoperatively, the patient was transferred to the interventional ward and received continuous urokinase infusion as part of systemic thrombolysis therapy. Two days later, follow-up venography still showed filling defects in the right superficial femoral and popliteal veins, prompting a second thrombus aspiration procedure. Another substantial volume of thrombus was removed, and the thrombolysis catheter was subsequently removed. The patient was then transferred to the general ward, where anticoagulation and antiplatelet therapy were continued.

### Follow-up

Upon discharge, the patient’s antithrombotic regimen consisted of rivaroxaban 15 mg twice daily and clopidogrel 75 mg once daily. On August 14, 2024, the patient reported no significant symptoms. A follow-up ultrasound showed partial recanalization of the DVT. Blood tests showed hs-TNI 0.009 ng/ml, NT-proBNP 221 pg/ml, and D-dimer 0.74 mg/L. The antithrombotic regimen was adjusted to rivaroxaban 20 mg once daily and clopidogrel 75 mg once daily. A follow-up ultrasound on September 4 confirmed thrombus recanalization, and the IVC filter was removed the next day. The patient was advised to continue regular follow-up.

## Discussion

PE and ACS share overlapping symptoms, such as chest pain and dyspnea, which can lead to misdiagnosis. Both conditions can present with non-specific signs, and elevated markers of myocardial injury may also occur in PE due to right ventricular strain. In ACS, sustained troponin elevation reflects left ventricular myocardial necrosis, whereas in PE, transient troponin rise is linked to right ventricular microinfarction. Similarly, NT-proBNP elevation in PE correlates with right ventricular strain, contrasting with its association with left heart failure in ACS. The predictive value of these biomarkers is supported by studies showing that a hsTnT cutoff ≥14 pg/mL in PE predicts adverse outcomes (OR = 4.97; 95% CI = 1.71–14.43; *P* = 0.003) (9), while elevated NT-proBNP strongly predicts right ventricular dysfunction (*P* < 0.001) and higher risks of in-hospital complications (OR = 6.8; 95% CI = 4.4–10) (10).

This challenge is heightened in patients with risk factors for both thromboembolic and cardiovascular diseases. In this case, the patient’s smoking, hypertension, and chronic kidney disease complicated the distinction between ACS and PE. Initial ECG changes and elevated troponins, usually linked to myocardial ischemia, suggested an acute cardiac event. CAG was promptly performed to rule out life-threatening causes, but the absence of significant coronary artery stenosis redirected the diagnostic focus to PE.

The diagnosis of PE often requires heightened clinical suspicion, especially when initial evaluations like CAG do not reveal cardiac causes of symptoms. The Wells and Geneva scores are widely used to assess PE probability and guide further testing (11). While the Wells score has high sensitivity, it has lower specificity, leading to more false positives in low-risk patients (12). The Geneva score, though less subjective, offers similar utility but may be slightly less sensitive in certain populations (13). It’s important to note that these tools are just part of the diagnostic process. In this case, although the patient had a low pretest probability based on both scores, the Pulmonary Embolism Rule-Out Criteria (PERC) couldn’t exclude PE, as the patient was over 50 years old (14). This illustrates the need for further diagnostic work-up even with low scores and highlights the importance of combining clinical judgment with comprehensive evaluation to avoid missing a PE diagnosis.

D-dimer is a fibrin degradation product often elevated in conditions involving significant clot formation and breakdown (15). Elevated D-dimer levels can occur in various conditions, including ACS, infections, malignancies, and trauma, reducing its utility as a standalone diagnostic marker (16). However, D-dimer is crucial in diagnosing PE, especially when combined with clinical pretest probability (C-PTP) assessments. A study indicated that among patients with low C-PTP and D-dimer levels below 1000 ng/mL, or moderate C-PTP with levels below 500 ng/mL, none of the 1,325 patients had thromboembolic complications during follow-up (17). This approach increased the percentage of patients avoiding chest imaging from 48.1 to 65.7%, representing a 33.9% relative reduction. These findings highlight that a normal D-dimer level can effectively rule out PE in low-to moderate-risk patients, while elevated levels require further investigation. In this case, the patient’s significantly elevated D-dimer raised suspicion of thromboembolism, confirmed by imaging showing DVT and PE. Thus, D-dimer testing should be routinely considered in patients with clinical ambiguity or thromboembolic risk factors to avoid missed PE diagnoses, especially when symptoms overlap with ACS.

The management strategies for PE and ACS differ significantly, particularly regarding antithrombotic therapy. While ACS treatment primarily relies on antiplatelet agents and reperfusion therapies (18), the cornerstone of PE treatment is anticoagulation, which prevents further clot formation and aids in resolving existing thrombi (11). When a clear diagnosis is uncertain, balancing the risks of bleeding against the benefits of preventing thromboembolic events is crucial. In patients with low bleeding risk, early anticoagulation initiation can be considered, especially if PE is strongly suspected. In this case, anticoagulation therapy was promptly started after CAG ruled out ACS, given the ongoing suspicion of PE. This early intervention is critical in reducing mortality and complications associated with PE.

This study provides novel insights into two critical challenges in cardiopulmonary medicine: (1) distinguishing low-risk PE from high-risk ACS and (2) refining therapeutic strategies for borderline-risk patients. First, we demonstrate that biomarker elevations (troponin and D-dimer) combined with echocardiographic evidence of right ventricular strain (moderate tricuspid regurgitation, Increased pulmonary artery pressure) may indicate clinically significant thrombotic burden in low-risk PE patients (PESI class I)—challenging sole reliance on clinical prediction scores. Second, we validate the role of aggressive mechanical interventions (dual thrombectomy + IVC filter) in patients with DVT, where anticoagulation monotherapy fails to address recurrent embolic risks. Third, we emphasizes a multidisciplinary approach for optimizing patient outcomes (19), especially in complex cases like this one. These findings underscore the necessity of physiologic risk assessment over static scoring systems and redefine therapeutic strategy for ostensibly “low-risk” PE patients to improve outcomes (20).

## Conclusion

Both PE and ACS are life-threatening conditions that require prompt diagnosis and treatment to prevent serious complications. This case report highlights the challenges in distinguishing between these two conditions, emphasizing the importance of thorough clinical evaluation when symptoms overlap. Clinicians should maintain a high index of suspicion for PE in patients presenting with ACS-like symptoms, particularly when initial cardiac investigations fail to reveal a clear ischemic cause. Timely recognition and appropriate management are essential to improve patient outcomes.

## Data Availability

The raw data supporting the conclusions of this article will be made available by the authors, without undue reservation.
